# Reliable reconstruction of HIV-1 whole genome haplotypes reveals clonal interference and genetic hitchhiking among immune escape variants

**DOI:** 10.1186/1742-4690-11-56

**Published:** 2014-07-04

**Authors:** Aridaman Pandit, Rob J de Boer

**Affiliations:** 1 Theoretical Biology and Bioinformatics, Utrecht University, Padualaan 8, 3584 CH Utrecht, The Netherlands

**Keywords:** Haplotype reconstruction, HIV-1 genome, Next generation sequencing, Clonal interference, Genetic hitchhiking

## Abstract

**Background:**

Following transmission, HIV-1 evolves into a diverse population, and next generation sequencing enables us to detect variants occurring at low frequencies. Studying viral evolution at the level of whole genomes was hitherto not possible because next generation sequencing delivers relatively short reads.

**Results:**

We here provide a proof of principle that whole HIV-1 genomes can be reliably reconstructed from short reads, and use this to study the selection of immune escape mutations at the level of whole genome haplotypes. Using realistically simulated HIV-1 populations, we demonstrate that reconstruction of complete genome haplotypes is feasible with high fidelity. We do not reconstruct all genetically distinct genomes, but each reconstructed haplotype represents one or more of the quasispecies in the HIV-1 population. We then reconstruct 30 whole genome haplotypes from published short sequence reads sampled longitudinally from a single HIV-1 infected patient. We confirm the reliability of the reconstruction by validating our predicted haplotype genes with single genome amplification sequences, and by comparing haplotype frequencies with observed epitope escape frequencies.

**Conclusions:**

Phylogenetic analysis shows that the HIV-1 population undergoes selection driven evolution, with successive replacement of the viral population by novel dominant strains. We demonstrate that immune escape mutants evolve in a dependent manner with various mutations hitchhiking along with others. As a consequence of this *clonal interference*, selection coefficients have to be estimated for complete haplotypes and not for individual immune escapes.

## Background

Inside every human host, HIV-1 embarks upon an arms race to evade the host’s immune responses [1,2]. A single (or a few) founder genetic lineage(s) establish the infection in the CD4+ cells localized in the mucosa at the port of entry [1,3-7]. HIV-1 then spreads to regional lymph nodes where it comes in contact with a large number of CD4+ T cells. Abundance of CD4+ T cells allows rampant viral replication resulting in millions of viral particles per ml of plasma at peak viremia, occurring ∼21–28 days post infection [3,8]. Interplay between HIV-1, target cell availability, and the host’s immune system during the acute phase of infection leads to a gradual decline of the viral load, eventually leveling off to a “set point” [2,5,9]. The decrease in the viral load following peak viremia is attributed to CD4+ T cell depletion, the immune responses of HIV-1 specific B cells, and cytotoxic T cells (CTLs). A high mutation rate and a large population size allow HIV-1 to rapidly evolve immune escape mutants [10,11], and strong CTL selection pressures confer fitness advantage to viral lineages that harbor CTL escape mutations [3,12,13]. The efficacy and breadth of cognate CTL responses increases the within-host HIV-1 diversity after peak viremia, and plays a major role in HIV-1 evolution during the acute phase of infection [5,9,14-19].

Longitudinally collected samples have been used to study the evolution of escape mutations, which can be used to estimate the selection pressure imposed by the corresponding CTL response [11,20,21]. The rate of escape is typically estimated using a logistic curve to model the replacement of wildtype epitope by escape variant [22]. Several escape mutations of the same epitope may occur simultaneously in different viral lineages, but such multiple escape pathways for a single epitope are generally modeled as a single escape event [12,17,18,23,24]. Escape mutations typically result in a fitness cost to the virus, and along different escape pathways the virus may face a different selection pressure by the CTLs [1,20,21,23,25]. Therefore, it was recommended to consider different escape pathways within the same epitope as separate escape events [23].

Escape mutations acquired in different epitopes are typically considered independent events. But in reality multiple epitopes may escape either in the same lineage, or in different lineages of the same viral population [1,11]. Viral lineages containing multiple escape mutations are expected to cause a selective sweep, reducing viral diversity and driving other lineages to extinction. The concurrent presence of different escape mutations in different viral lineages may result in competition between them in their race towards fixation [23,26,27] via *clonal interference*[28,29]. Due to clonal interference, two beneficial mutations residing in two different lineages out-compete each other as well as the wild-type when one approaches fixation. Additional beneficial mutations can be sequentially acquired, ultimately resulting in escape mutations in multiple epitopes [17,21,23,27]. As a result of clonal interference, epitope escape mutations may also drive several non-epitope mutations, co-occurring in the same viral lineage, to fixation via *genetic hitchhiking*[26,30,31]. Clonal interference has recently been shown to play an important role in the evolution of influenza [26,27]. One would expect it to also influence the evolution of HIV-1, but as yet there is no evidence for this.

To study the role of clonal interference and genetic hitchhiking in HIV-1 evolution, one requires long, or ideally complete, genome sequences with high coverage from multiple time points. The availability of longitudinally sampled single genome sequences is rather limited, and next generation sequencing (NGS) is economically a more feasible alternative for single genome sequencing [1,7,32]. One large NGS dataset was generated by Henn et al. [11], when they performed NGS on complete HIV-1 genomes from 6 longitudinal samples in one patient (subject 9213). NGS allowed Henn et al. [11] to study the dynamics of immune escape mutations in individual epitopes. However, due to the short sequencing read lengths of about ∼ 400 bp, Henn et al. [11] were not able to distinguish whether multiple epitopes escape simultaneously in the same haplotype,or independently in distinct viral haplotypes. We here attempt to reconstruct whole genome haplotypes from this data. This is challenging due to two reasons [33,34]. First, a variety of sequencing and PCR errors are incorporated in sequencing reads, which require robust error correction techniques to identify the true mutations [33,35,36]. A second problem concerns the determination of contiguity of sequencing reads generated from a single viral genome haplotype. Differences in prevalence of the genome haplotypes, genetic distances between polymorphic sites, and the amount of sequencing errors, are important factors influencing the reliability of haplotype reconstruction. A number of algorithms have been developed for viral haplotype reconstruction [33,34,37,38], and among them PredictHaplo has been shown to perform at high precision in reconstructing gene haplotypes at a high sequence divergence [34,39].

We here demonstrate that reconstruction of whole genome HIV-1 haplotypes from NGS datasets is feasible using a read clean-up step before haplotype prediction with PredictHaplo. The reconstructed genome haplotypes provide important insights into the evolutionary dynamics of the viral quasispecies, as we show that the epitope escape dynamics are influenced by the strength and breadth of CTL selection, clonal interference, and genetic drift.

## Results and discussion

### Testing the feasibility of haplotype reconstruction using simulated datasets

Before reconstruction of HIV-1 whole genome haplotypes from next generation sequencing reads, we first tested the feasibility of the haplotype reconstruction pipeline using *in silico* datasets. We generated six *in silico* HIV-1 populations with nucleotide diversities (1, 2, 4 and 10%) [11,40,41] and frequency distributions (uniform or log-normal, see Methods). For each dataset, ART_454 software [42] was used to generate *in silico* next-generation sequencing reads. Each simulated population consisted of 9 master HIV-1 genomes that were 8800 bp long. For the 4 data sets with uniform frequency distributions (data sets U1, U2, U4 and U10, respectively), our pipeline reconstructed 9 haplotypes from the short reads artificially generated from the true 9 *in silico* genomes (see Methods). The reconstruction was accurate with a Hamming distance of just 1 to 9 nucleotides from the true genomes (Table 1).

**Table 1:** Haplotype reconstruction performance on simulated datasets

**Dataset**	**Nucleotide**	**No. of haplotypes**	**Mean Hamming**	**MHD of top 4**	**Range Hamming**
	**diversity**	**reconstructed**	**distance (MHD)**	**haplotypes**	**distance**
U1	1	9	6	6	(6-6)
U2	2	9	4.33	2.25	(1-9)
U4	4	9	4.33	2	(2-9)
U10	10	9	4.66	2.25	(1-9)
L1	1	6	49.5	28.25	(11-93)
L2	2	5	46.2	23	(7-139)
L4	4	9	158.44	11	(1-427)
L10	10	7	283.85	65.25	(46-706)
LHV	1+	9	20.66	13.5	(13-37)

Simulated datasets were named according to the frequency distribution of their population and their nucleotide diversity. Populations with uniform and log-normal frequency distributions are labeled as U and L, respectively, followed by a number denoting the percentage nucleotide diversity between genomes. In all cases there were 9 “true” genomes.

The accuracy decreased when the population structure was changed to a log-normal frequency distribution (data sets L1, L2, L4 and L10, respectively). For instance, for the L4 dataset with 4% nucleotide diversity, the 9 reconstructed haplotypes had a Hamming distance of 1 to 427 nucleotides from the corresponding true genomes (Table 1). Closer examination, however, revealed that the four most prevalent (>4%) haplotypes were accurately reconstructed, with Hamming distances of 1, 1, 19 and 23. Additionally, most of the errors were limited to either the start or end of the genomes, where the simulated coverage was low. Ignoring the initial and final 20 bases of the haplotype sequences, the four most prevalent haplotypes were identical to their closest true genomes.

Some regions of HIV-1 genome are know to have higher mutation rate than others [43]. To model the variability in mutation rate in HIV-1, we simulated a viral population (LHV) with genomes containing 3 hypervariable regions (see Methods). The 9 reconstructed genomes from the LHV data set had a Hamming distance of 13 to 37 nucleotides from the corresponding true genomes (Table 1), whereas the four most prevalent haplotypes were reconstructed at a Hamming distance of 13, 13, 13 and 15. For all data sets, the predicted frequencies of the reconstructed haplotypes matched those of the true genomes (results not shown).

**Figure 1:**
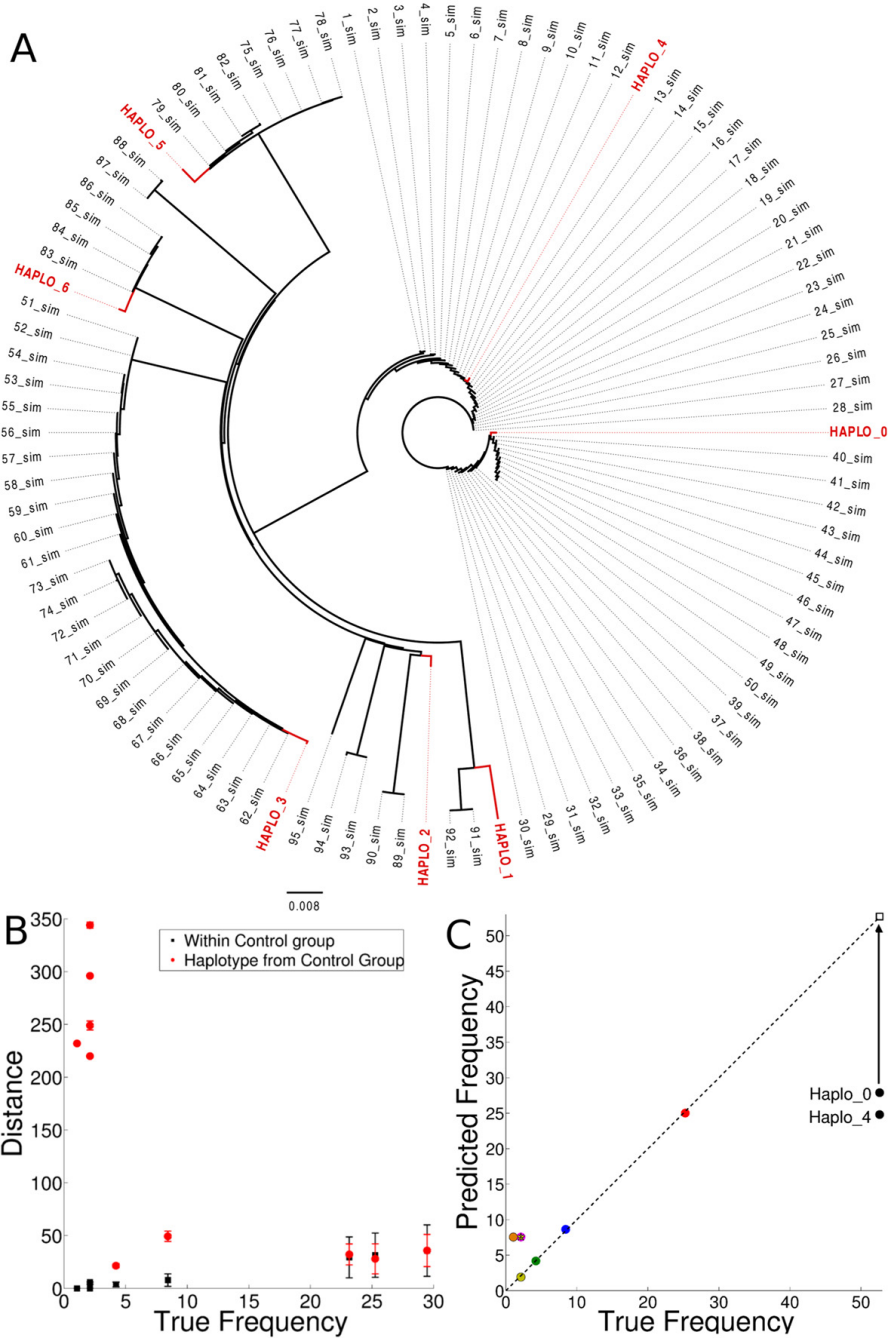
**Accuracy of reconstructed haplotypes from simulated HIV-1 quasispecies (LQ4_1).** We simulated HIV-1 population (LQ4_1; Table 2) with 9 quasispecies consisting of 50, 24, 8, 4, 2, 2, 2, 2 and 1, sequences respectively. The genome sequences within each quasispecies contained at most 0.1 *%* nucleotide diversity (see Methods). **(A)** Seven reconstructed whole genome haplotypes (red) were aligned with the 95 simulated genomes. Dark lines represent the branches of the phylogenetic tree (the dotted lines pointing outwards provide better visualization). **(B)** Average Hamming distance within each quasispecies (black), and between the reconstructed haplotype and the quasispecies at minimum Hamming distance (red). Because one haplotype can represent multiple quasispecies, we used the phylogenetic tree to determine the genomes represented by a haplotype. Markers represent the mean Hamming distance and error bars represent 1 standard deviation. **(C)** The haplotype frequencies (vertical axis) predicted from haplotype reconstruction pipeline are plotted against the true quasispecies frequencies (horizontal axis). The dominant quasispecies was reconstructed as two haplotypes (Haplo_0 and Haplo_4) and summation of their predicted frequencies is indicated by an open square. The haplotype Haplo_2 (predicted frequency of 7.55%) represents four low prevalence quasispecies: one quasispecies with true frequency of 1.05% (orange circle) and three with true frequencies of 2.1% (see the overlapping points next to orange circle). The points lying on the dashed diagonal line represent perfect haplotype frequency predictions.

In reality, HIV-1 is known to have a complex population structure involving multiple co-occurring quasispecies [44]. To mimic this, we simulated a more complex population (*LQ4_i*) consisting of 9 master HIV-1 genomes corresponding to 9 quasispecies, each surrounded by a varying number of closely related genomes (Table 2 and Figure 1A; see Methods). This was simulated a 100 times, and for each simulation a minimum of 5 and a maximum of 8 haplotypes were reconstructed (Table 2). The 4 most abundant haplotypes represented the 4 most prevalent quasispecies in the population, and these were more accurately reconstructed than the low frequency haplotypes. Most sequence errors were found in the initial and final 50 bp of the reconstructed haplotypes, exclusion of which resulted in a large reduction in the Hamming distance between the reconstructed haplotypes and the true genomes, especially for the prevalent haplotypes (Table 2).

**Table 2:** Haplotype reconstruction of quasispecies datasets

**Dataset**		**No. of haplotypes**		**Mean Hamming**	**MHD of top 4**	**MHD of**
		**reconstructed**		**distance (MHD)**	**haplotypes**	**top 4: pruned**
	**Mode**	**Min**	**Max**			
LQ4_1 to LQ4_100	6	5	8	75.76	13.63	4.94

Haplotype reconstructions from populations having a simulated viral quasispecies structure. Nine quasispecies were present in each simulated population and the diversity between them was 4% (see Methods). The pruned haplotype genomes were created by excluding the initial and final 50 bp from the reconstructed haplotypes to avoid errors due to low read coverage. A 100 simulations were performed.

We now present a detailed analysis of LQ4_1, as a representative example (the 9 other simulations were similar). The five most prevalent haplotypes (haplo_0, haplo_3, haplo_4, haplo_5 and haplo_6; see Figure 1) were reconstructed at a Hamming distance of 8 to 46 nucleotides from their closest sequences (Table 2), and were reconstructed as a “consensus genome” of their corresponding quasispecies (Additional file 1: Figure S1). However, the most abundant quasispecies containing 50 simulated genomes, was reconstructed as two haplotypes, i.e. haplo_0 and haplo_4, each “representing” 22 and 28 simulated genomes (Figure 1A). The remaining two haplotypes (haplo_1 and haplo_2) were reconstructed as consensus genomes of the five rare (frequencies <4%) quasispecies (Figure 1A). Variation in the Hamming distance between the reconstructed haplotype and genomes of the corresponding quasispecies was comparable to the intrinsic variation within the quasispecies (Figure 1B). The dominant quasispecies were accurately reconstructed, but the less frequent quasispecies were reconstructed at a much higher Hamming distance (Figure 1B). Since haplotypes are reconstructed as a consensus of each quasispecies (Additional file 1: Figure S1), variations between the reconstructed haplotypes and their true genome can at least partly be attributed to the viral quasispecies population structure. The predicted prevalence of the dominant haplotypes was comparable to the true frequencies of the corresponding quasispecies (Figure 1C). Summarizing, these simulations suggest that the haplotype reconstruction pipeline works with high fidelity for complete HIV-1 genome haplotype reconstruction using 454 sequencing reads with realistic nucleotide diversities of 1 to 10%, and genotype frequencies larger than 4%.

We have used PredictHaplo to reconstruct genome haplotypes. Several other algorithms are available for haplotype reconstruction [34,37-39]. To test the performance of these algorithms we also reconstructed haplotypes using two other algorithms, QuRe and ShoRAH. For the *in silico* HIV-1 data sets (given in Tables 1 and 2), each containing 9 true genomes, ShoRAH reconstructed ≥187 haplotypes with a ≥117.80 mean Hamming distance for each data set (Table 3). For the same *in silico* HIV-1 data sets (Tables 1 and 2), QuRe reconstructed ≥21 haplotypes with a ≥211.18 mean Hamming distance for each data set (Table 3). Corroborating a previous study by Schirmer et al. [34], we see that ShoRAH and QuRe overestimate the number of haplotypes and have lower precision to predict “true” haplotypes (Table 4). PredictHaplo reconstructs haplotypes more reliably than both ShoRAH and QuRe even if we only consider the four most prevalent haplotypes (Tables 1, 2, and 3). Due to the large number of spurious haplotypes predicted by ShoRAH and QuRe, the predicted haplotype frequencies do not match with the true frequencies (results not shown), whereas the haplotype frequencies predicted by PredictHaplo were accurate (as shown in Figure 1C). Note that previous studies have shown that both QuRe and PredictHaplo reconstruct shorter haplotypes (length ∼1400 bp) at high precision [39]. Since the aim of our study was not to benchmark algorithms but to study the evolutionary dynamics of immune escape mutations. We continue with PredictHaplo as it produced the lowest number of spurious whole genome haplotypes, and accurately predicted the haplotype frequencies. The other algorithms may prove more apt to address different questions.

**Table 3:** Comparison of haplotype reconstruction algorithms

**Dataset**	**ShoRAH**			**QuRe**		
	**No. of haplotypes**	**MHD**	**MHD: top 4**	**No. of haplotypes**	**MHD**	**MHD: top 4**
	**reconstructed**			**reconstructed**		
U2	187	117.80	0	104	228.66	168.50
U4	201	201.49	0	146	416.38	230.50
U10	200	315.515	1	86	607.10	83.75
L2	210	217.47	186	21	260.24	245.25
L4	205	577.97	336.25	48	565.29	510.75
L10	344	1182.13	886.25	NA	NA	NA
LQ4_1	200	220.485	60.75	27	211.18	103

Simulated viral populations given in Tables 1 and 2 were also analyzed by two alternative haplotype reconstruction algorithms, i.e., ShoRAH and QuRe [33,37,38]. All datasets contained 9 “true” genomes. NA indicates that QuRe was terminated after two weeks of CPU run time (3.1 GHz 16 core Intel Xeon processor, 128GB RAM).

**Table 4:** Precision of haplotype reconstruction algorithms

**Algorithm**	**U2**	**U4**	**U10**	**L2**	**L4**	**L10**	**LQ4_1**
PredictHaplo [53]	1	1	1	0.60	0.44	0	0.57
ShoRAH [38]	0.10	0.18	0.39	0	0	0	0
QuRe [37]	0	0	0.01	0	0	NA	0

We calculated the precision by taking the ratio between the number of accurately reconstructed haplotypes over the total number of reconstructed haplotypes. An haplotype was considered to be accurately reconstructed if the Hamming distance to the “true” genome was ≤25. Recall values are not listed as most of them are 0 for ShoRAH and QuRe.

### Experimental validation of the reconstructed haplotypes

The same methodology is now applied to the NGS data from a patient described in Henn et al. [11]. In brief, this patient presented during acute infection with a viral load of 9.3×10^6^ copies/ml, and henceforth this is referred to as day 0. Six longitudinal serum samples were collected at day 0, 3, 59, 165, 476 and 1543 post presentation by Henn et al. [11]. The peak viral load was observed at day 3. The first four samples represent the acute phase dynamics, while the last two samples fall in the chronic phase of infection. Deep sequencing (Roche 454 Genome Sequencer FLX Titanium) was performed on each sample using four overlapping PCR amplicons spanning the complete protein coding HIV-1 genome. The average fold sequence coverage for the samples from day 0, 3, 59, 165, 476 and 1543 was 667.7, 724.4, 750.5, 299.7, 227.6 and 540.7, respectively. CTL epitope escapes restricted by subject’s HLA alleles (A01, A24, B38, B44 and Cw04) were studied using a local read analysis, and confirmed using IFN-gamma ELISPOT assays. The most dominant CTL responses at day 59 were directed against the Nef-RW8 and Vif-WI9 epitopes. To validate the sequencing reads, Single Genome Amplification (SGA) was performed for *vif* gene sequences from day 59. The public availability of this data allowed us to test the validity of our whole genome haplotype reconstruction pipeline.

#### ***Validation of reconstructed haplotypes by SGA***

**Figure 2:**
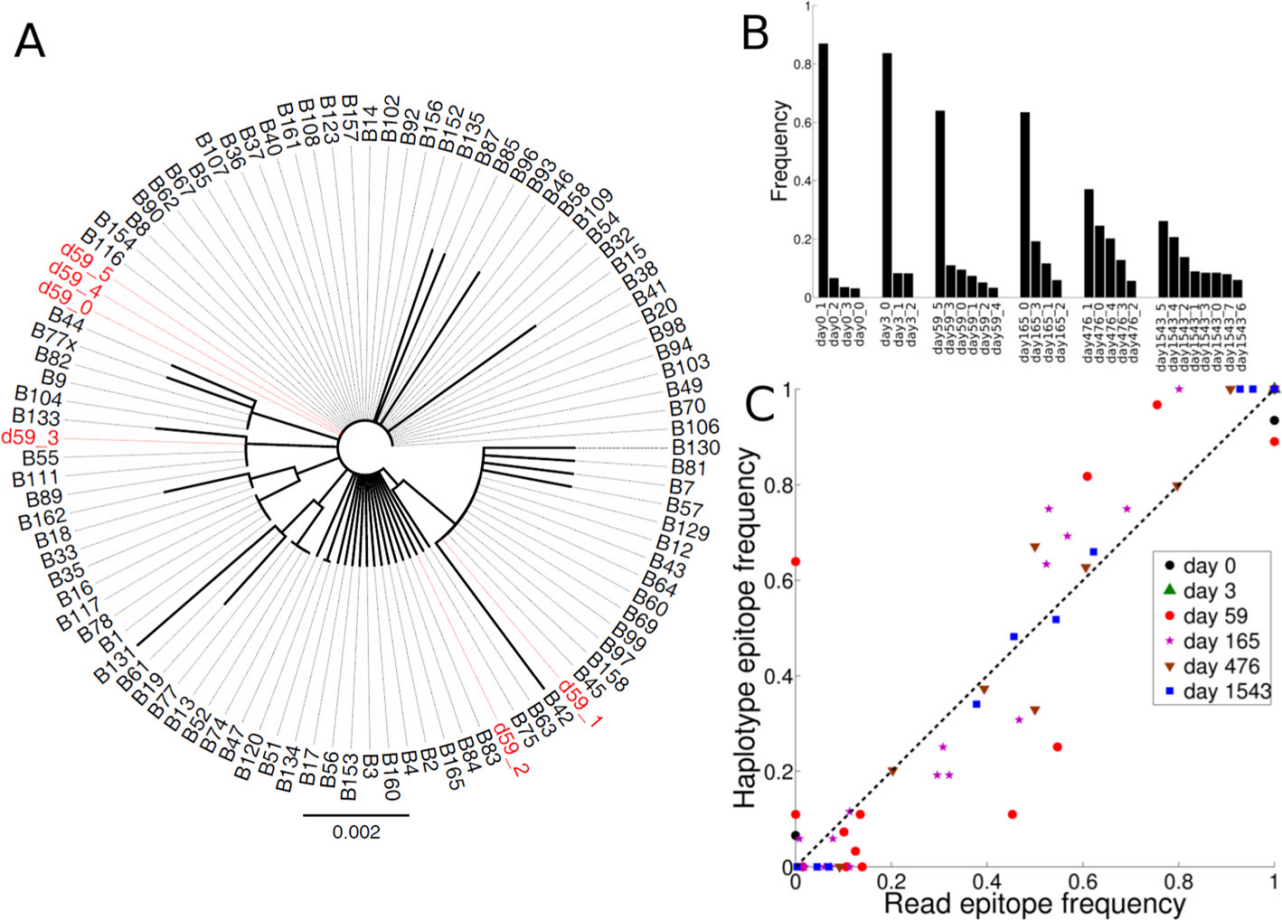
**Validation of reconstructed haplotypes from subject 9213. (A) ** Neighbor-joining phylogenetic tree generated from the global alignment of the *vif* genes extracted from the 6 haplotypes reconstructed from the day 59 data (red) and the SGA *vif* gene sequences (black) obtained from [11]. **(B)** The predicted haplotype frequencies for subject 9213 plotted for each sample (day 0, day 3, day 59, day 165, day 476, and day 1543). Note that the variation in read coverage (667.7, 724.4, 750.5, 299.7, 227.6 and 540.7, respectively) does not seem to influence the number of predicted haplotypes. **(C)** Comparison of the epitope frequencies estimated from reconstructed haplotypes and those from the local analysis of sequencing reads described in [11]. Markers represent different days. Variants of each epitope are represented as different observations. Several epitope variants with 100% read frequency and a 100% predicted frequency in reconstructed haplotypes appear as overlapping points in the upper right corner on the diagonal. The overlapping points were not considered for the correlation analysis. The Spearman’s rank correlation coefficient between predicted epitope frequencies extracted from complete genome haplotypes and the local read frequencies was *ρ* = 0.85 (p-value < 10^-15^).

As the first validation step, the 95 SGA *vif* sequences from day 59 [11] were aligned with the 6 *vif* gene sequences extracted from the whole genome haplotypes reconstructed using the NGS data from day 59. Phylogenetic analysis showed that five reconstructed *vif* sequences were identical to 48 out of 95 SGA *vif* sequences (Figure 2A). Of the remaining SGA sequences, 11 were found in clades that were represented by at least one reconstructed haplotype (e.g., B7, B42, B57, B81 and B130 were represented by d59_1). 16 low frequency SGA variants, depicted as singletons in Figure 2A, were represented by one haplotype (d59_2). Thus, 75 of the 95 observed SGA sequences were represented by 6 reconstructed genome haplotypes. The remaining 20 SGA sequences were prevalent at low frequencies, and were not reconstructed as a unique haplotype. Even though we reconstruct whole genome haplotypes, we apparently detect haplotypes with mutations in just a few bases within a region of 600 bp (spanning at least 2 reads), confirming that global haplotypes accurately capture local features.

#### ***Temporal variations in haplotype prevalence frequencies***

Next to reconstructing the sequences, we should also be able to predict the correct genotype frequencies of the haplotypes. Due to transmission bottlenecks, HIV-1 infection is typically established by a single (or a few) genetic lineage(s) [1,4-6,23], and the HIV-1 population does not exhibit much diversity until the peak viremia [11]. One should therefore expect one (or a few) dominant HIV-1 haplotype(s) before and around the time viremia peaks. Around the peak viremia, immune selection leads to diversification, i.e. a decrease in the frequency of the dominant quasispecies, and an increase in frequency of other quasispecies in the population [1,23]. Reconstructing the haplotypes from every sample, we found that the number of reconstructed haplotypes varied from 3 to 8 (Figure 2B). Although the viral population at day 0 (i.e., before peak viremia) consisted of 4 haplotypes, only one haplotype (d0_1) was present at a high frequency (∼87%), confirming that this infection was established by a single founder virus lineage. At day 3 (around peak viremia), the same dominant haplotype (d3_0, identical to d0_1) was present at a similar high frequency (∼83.6%). Diversification led to a decrease in the frequency of dominant haplotypes at day 59 and 165. Several haplotypes with comparable frequencies were reconstructed for day 476 and 1543, indicating diversification and expansion of multiple lineages during the early chronic phase of infection (Figure 2B). The reconstructed haplotypes thus suggest a realistic scenario where the infection in subject 9213 was started by a single haplotype, followed by an increase in viral diversity resulting in the establishment of multiple co-dominant viral quasispecies.

#### ***Validation of whole genome haplotypes using predicted epitope frequencies***

As a third validation, we compared the frequencies of the CTL epitopes, obtained by summation of the predicted frequencies of those haplotypes containing the CTL epitope, with the observed read frequencies described in Henn et al. [11]. The average nucleotide diversity between whole genome haplotypes from the same sample was 128 nucleotides. As the epitope escape mutations comprise only a small fraction of all the polymorphic sites used to infer the whole genome haplotypes, this comparison is a fairly independent validation. Since it is trivial that the epitopes containing no variation in a given sample will always exhibit 100% read and haplotype frequencies, they were not considered for the correlation analysis. The predicted frequencies of the epitopes were accurate and tightly correlated with the observed frequencies (Spearman’s *ρ *= 0.85, p-value < 10^-15^) (Figure 2C). Some of the low frequency epitope variants were not predicted by haplotype reconstruction, and they could either be sequencing errors, or true variants not containing sufficient contiguous reads to allow their reconstruction. The Nef RW8-T5M, the Gag A01-R6K and the Gag A01-E3A escape mutations, which were not detected by the local read analysis of Henn et al. [11], were predicted at ≥ 6.5%, frequency on the day 0, and 59 haplotypes (red, and black circles on vertical axis, Figure 2C). Henn et al. [11] performed several additional clean-up steps (read phasing, read profiling, and removal of reads partially spanning an epitope), which apparently discarded the reads containing these Nef and Gag variants. Because the Nef RW8-T5M and the Gag A01-R6K mutations were present at subsequent time points [11], we think their detection by the pipeline is correct. To summarize, we can correctly predict the epitope frequencies by reconstructing whole genome haplotypes.

### Biological results

#### ***Phylogenetic analysis of reconstructed haplotypes***

**Figure 3:**
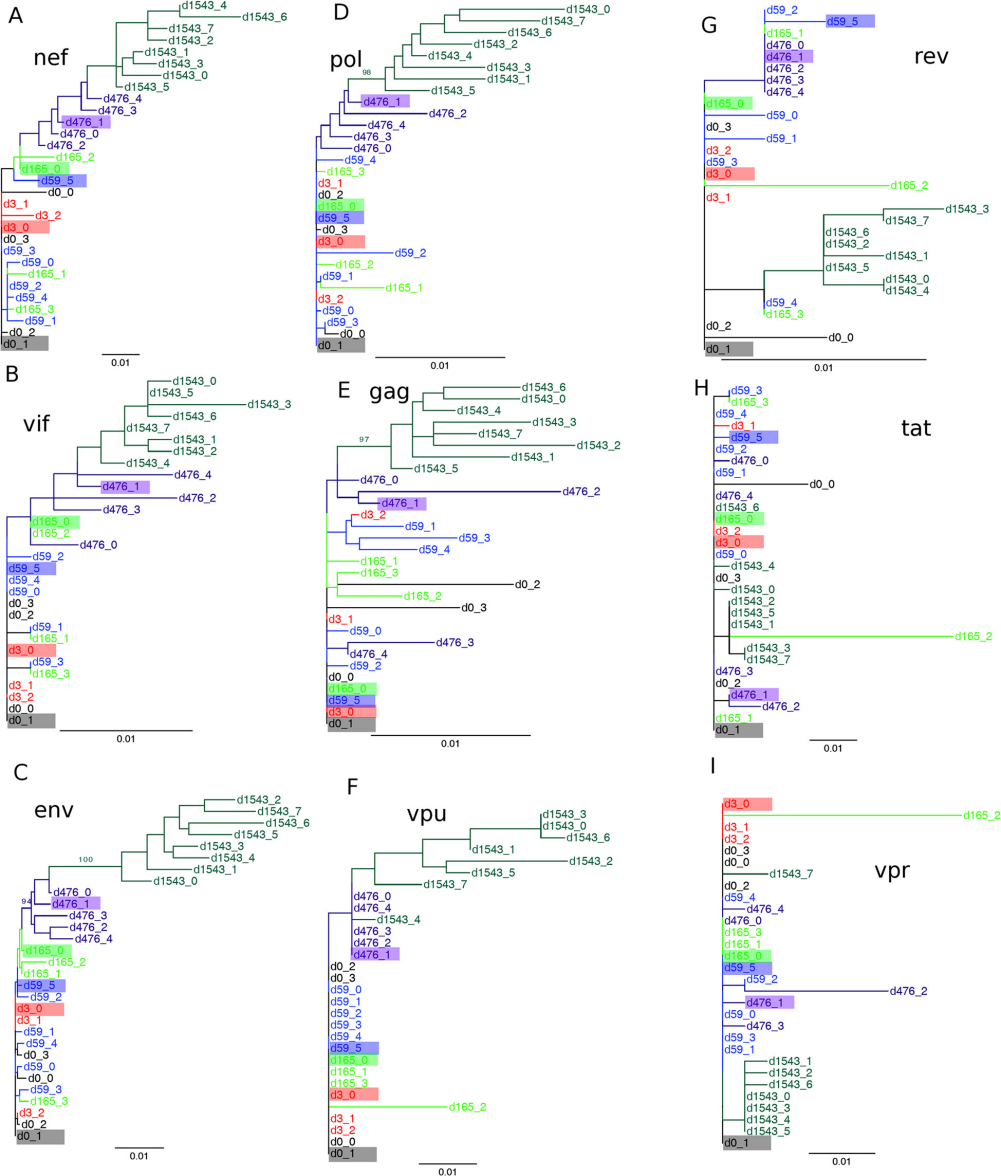
**Phylogenetic trees of the reconstructed HIV-1 genes extracted from genome haplotypes. **The phylogenetic trees generated from the nucleotide sequences of the **(A)***nef*, **(B)***vif*, **(C)***env*, **(D)***pol*, and **(E)***gag* genes, that are targeted by one or more CTLs [11] exhibit a strong to intermediate signal for temporal selection. The *vpu ***(F)**, *rev ***(G)**, *tat ***(H)**, and *vpr ***(I)** genes exhibit weak or no signal for temporal selection in their phylogenetic trees. The maximum likelihood (ML) phylogenetic trees were reconstructed with PHYML using the GTR nucleotide substitution model, gamma distributed rate variation across sites and 6 substitution rate categories (see Methods for details). 100 bootstrap replicates were performed and support values of > = 90 are shown on the corresponding branch. Colors denote different temporal haplotypes: day 0 (grey), day 3 (red), day 59 (blue), day 165 (green), day 476 (purple) and day 1543 (teal). The dominant haplotype for each sample is highlighted by a colored box.

**Figure 4:**
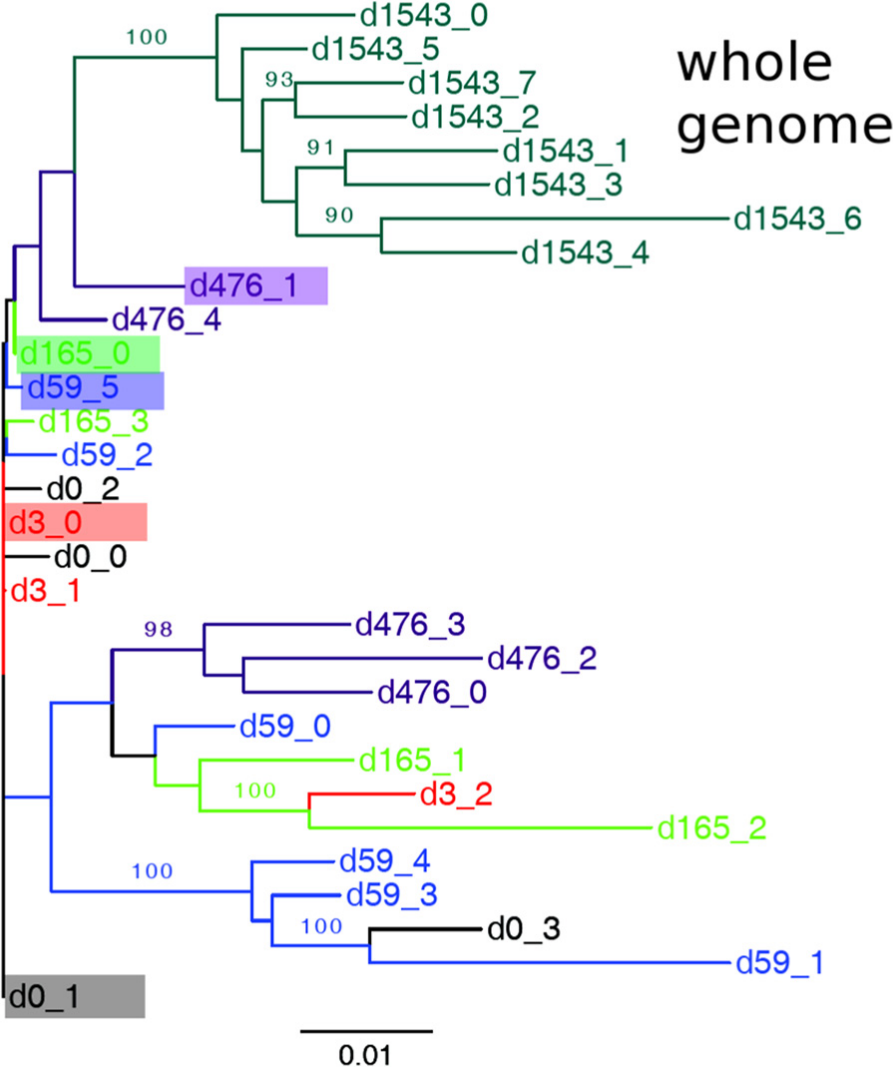
**Phylogenetic tree for complete genome haplotypes. **A whole genome nucleotide phylogenetic tree was generated using the reconstructed haplotypes. The ML phylogenetic tree was made with PHYML using the GTR nucleotide substitution model, gamma distributed rate variation across sites and 9 substitution rate categories (see Methods for details). 100 bootstrap replicates were performed and support values of > = 90 are shown on the corresponding branch. Colors as in Figure 3.

A hallmark of HIV-1 evolution during the acute phase of infection is the selection by the immune system [1,45]. The successive replacement of viral haplotypes in the phylogenetic trees in Figure 3 reveals that HIV-1 in subject 9213 also underwent a selection driven evolution. The *nef*, and *vif* genes, which are targeted by dominant CTL responses [11], and the *env* gene targeted by 3 sub-dominant CTL responses [11], exhibit a strong temporal phylogenetic signal of successive replacement of dominant haplotypes (Figure 3A-C). Strong temporal selection causes all day 1543 haplotypes to form a monophyletic clade diverging from the clade containing the dominant haplotype at day 476 (d476_1) (Figure 3A-C). Using codon selection analysis we found that 11 codons in *nef*, 4 codons in *vif* and 50 codons in *env* were under positive selection (see Methods). The *gag*, and *pol* genes targeted by sub-dominant CTL responses exhibit intermediate phylogenetic signals for temporal selection (Figure 3D and E). The trees of the remaining genes (*rev*, *tat*, *vpr*, and *vpu*) did not reveal immune selection driven evolution (Figure 3F-I). Codon selection analysis showed that no codon was under positive selection in *tat*, *vpr*, and *vpu*; whereas 1 codon in *rev* was under positive selection (see Methods). The complete genome phylogeny captured the temporal dynamics exhibited by the genes under identified CTL selection, and showed similar successive replacement of dominant haplotypes (Figure 4). Thus, phylogenetic analysis indicates temporal replacement of the dominant quasispecies, and that this signal is most evident in the genes targeted by the CTLs.

#### ***Clonal interference and epitope escape***

**Figure 5:**
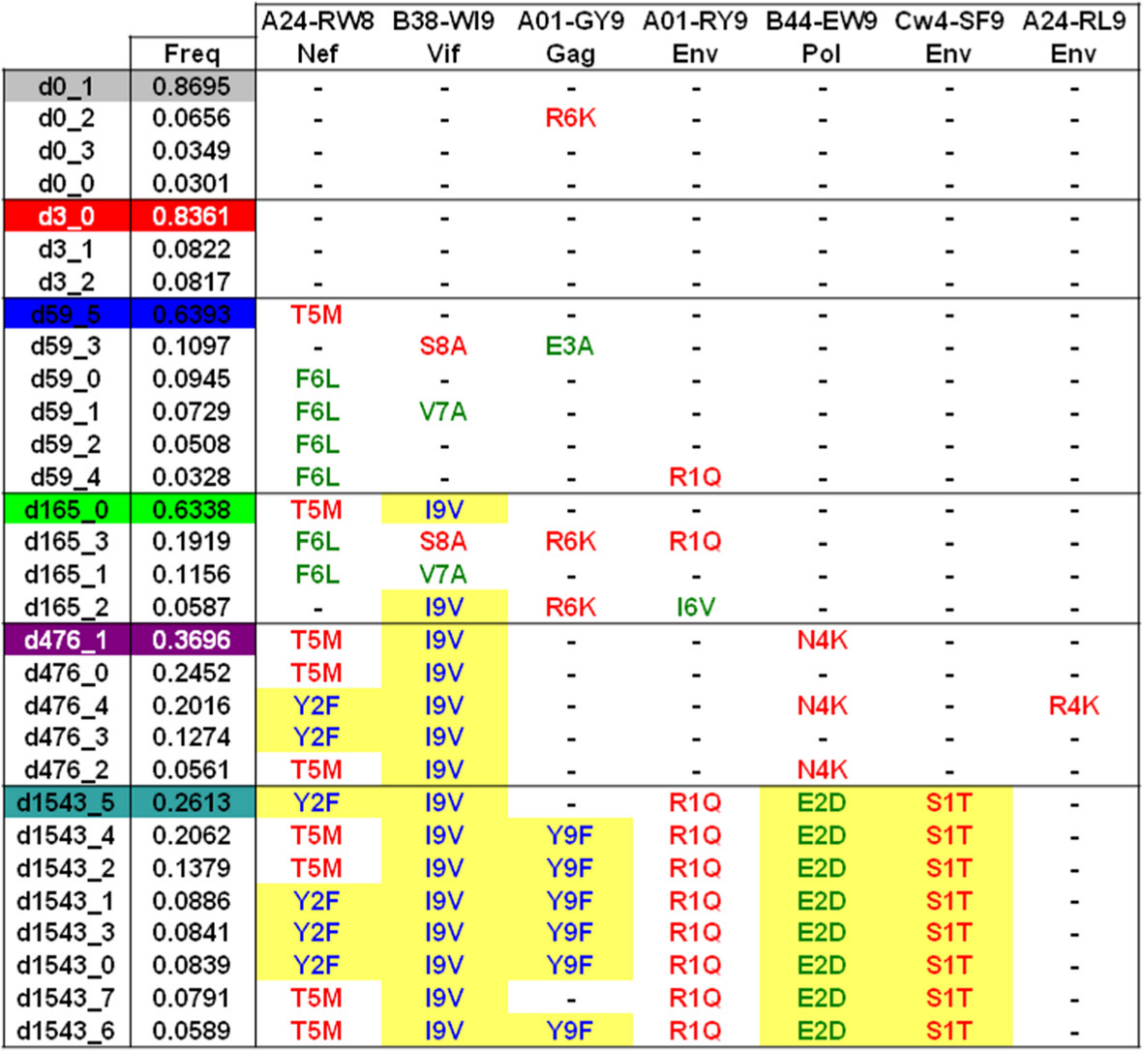
**Epitope escapes in complete genome haplotypes.** The escape mutations acquired in the seven epitopes identified by Henn et al. [11] are given per reconstructed haplotype for each time point. Haplotypes reconstructed from each temporal sample are given in a descending order of their frequencies. The dominant haplotype for each time point is depicted by the same colors as in Figure 3. Multiple escapes for the same epitope are depicted by different colors. Escape mutations for each epitope are denoted by a 3-letter code with the first letter as the original epitope amino acid, second letter as the position in the epitope and the third letter as the mutated amino acid. Mutations at anchor residues are highlighted.

Studying the 7 identified CTL epitopes in the whole genome haplotypes reveals that the virus explores multiple escape pathways in most epitopes. Different combinations of escape mutations were found in different haplotypes from the same population (Figure 5). The presence of different combinations of escape mutations in each haplotype should confer different fitness advantages to them resulting in clonal interference between the viral haplotypes. This illustrates that the selective advantage of escape mutations has to be considered in combination. The population at day 59 contained 6 escape mutations distributed over 4 different epitopes, 2 in Nef A24-RW8, 2 in Vif B38-WI9, 1 in Gag A01-GY9 and 1 in Env A01-RY9 (Figure 5). Interestingly, haplotype d59_3 containing 2 of the CTL escape mutations disappeared before day 165, while d59_5, which was the dominant haplotype at day 59 with only one escape mutation survived, and additionally evolved an anchor position escape mutation in the Vif B38-WI9 epitope by day 165. Interestingly, haplotype d165_3 contained 4 epitope escapes and was not selected over time, whereas haplotype d165_0 containing two escape mutations was selected (Figure 5).An epitope typically contains two anchor positions that are essential for binding the MHC molecule. An epitope with an anchor position escape therefore results in the evasion from all potential CTL responses, as the epitope is no longer presented by the MHC molecule. This should confer a higher fitness compared to non-anchor position escapes which can be targeted by other CTL populations. This could explain why haplotype d165_0 with an anchor position escape (I9V) for the dominant Vif epitope was selected over haplotype d165_3 with the non-anchor S8A escape (Figure 5). Additionally, the strength of CTL selection may influence the fate of other epitope escape mutations. The fixation of haplotype d165_0, containing the escape mutations Vif B38-I9V and Nef A24-T5M from two dominant CTL responses, could therefore explain why the Gag A01-R6K and Env A01-I6V escape mutations in the d165_2 haplotype disappeared from the population (Figure 5). Thus, several factors like the strength of each CTL response, breadth of CTL responses, and genetic drift, together appear to determine the relative fitness of a haplotype. Most importantly, beneficial epitope escape mutations disappear due to higher relative fitness of other haplotypes present in the viral population, emphasizing that clonal interference plays an important role in acquisition of epitope escape mutations by HIV-1.

#### ***Selection of haplotype genomes***

**Figure 6:**
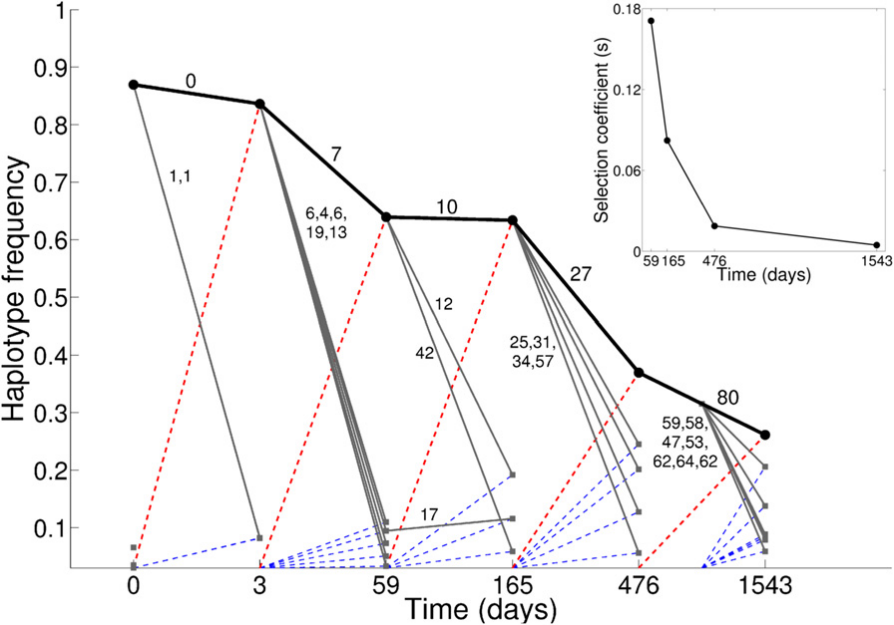
**Selection dynamics of complete genome haplotypes.** Dominant haplotypes for each sample are denoted by black circles, and the less abundant haplotypes are denoted by grey squares. The dominant and less abundant haplotypes are connected to those haplotypes from the previous time point having the minimum non-synonymous Hamming distance (mentioned above each edge). The less prevalent haplotypes at day 1543 were closer to the dominant haplotype at the same day compared to any haplotype present at day 476, and thus are shown to have a predecessor at an intermediate time. The rate of replacement by a haplotype genome is represented by dashed lines: in red for dominant haplotypes, and in blue for non-dominant haplotypes. Note that the horizontal axis has no true time scale. Selection coefficients for dominant haplotypes are shown in the inset. Selection coefficients for the dominant haplotype genomes were calculated using the predicted haplotype frequency at time *t* and its frequency at preceding time point *t*-1 using the method described in [22]. The mutant was considered to have a prevalence of 3% at the preceding time point (because we cannot reliably detect haplotypes below a 3% frequency).

Figure 6 depicts the temporal dynamics of the reconstructed HIV-1 haplotype genomes. The dominant (black circle) and other less abundant (gray squares) haplotypes at each time point were connected to the preceding haplotype with the minimal non-synonymous Hamming distance (mentioned above each line in Figure 6). The haplotypes were thus connected with the preceding haplotypes based on their sequence similarity. In all six samples, the dominant quasispecies seeded the subsequent dominant quasispecies that was generally located at a lower mutational distance, whereas the other less prevalent quasispecies were located at a higher mutational distance from the ancestral haplotype (Figure 6). The sequence of the dominant haplotype did not change between day 0 and day 3, but differed from its predecessor at all subsequent time points (Figure 6). The rate at which the dominant haplotype is replaced by the subsequent haplotype (denoted by red dashed lines in Figure 6) decreased over time from 0.17 to 0.005 per day (Figure 6, inset). The rates of replacement calculated using whole genome haplotypes better represent the evolution of HIV-1 under immune selection than the set of escape rates estimated on the basis of individual epitopes [11].

#### ***Genetic hitchhiking***

**Figure 7:**
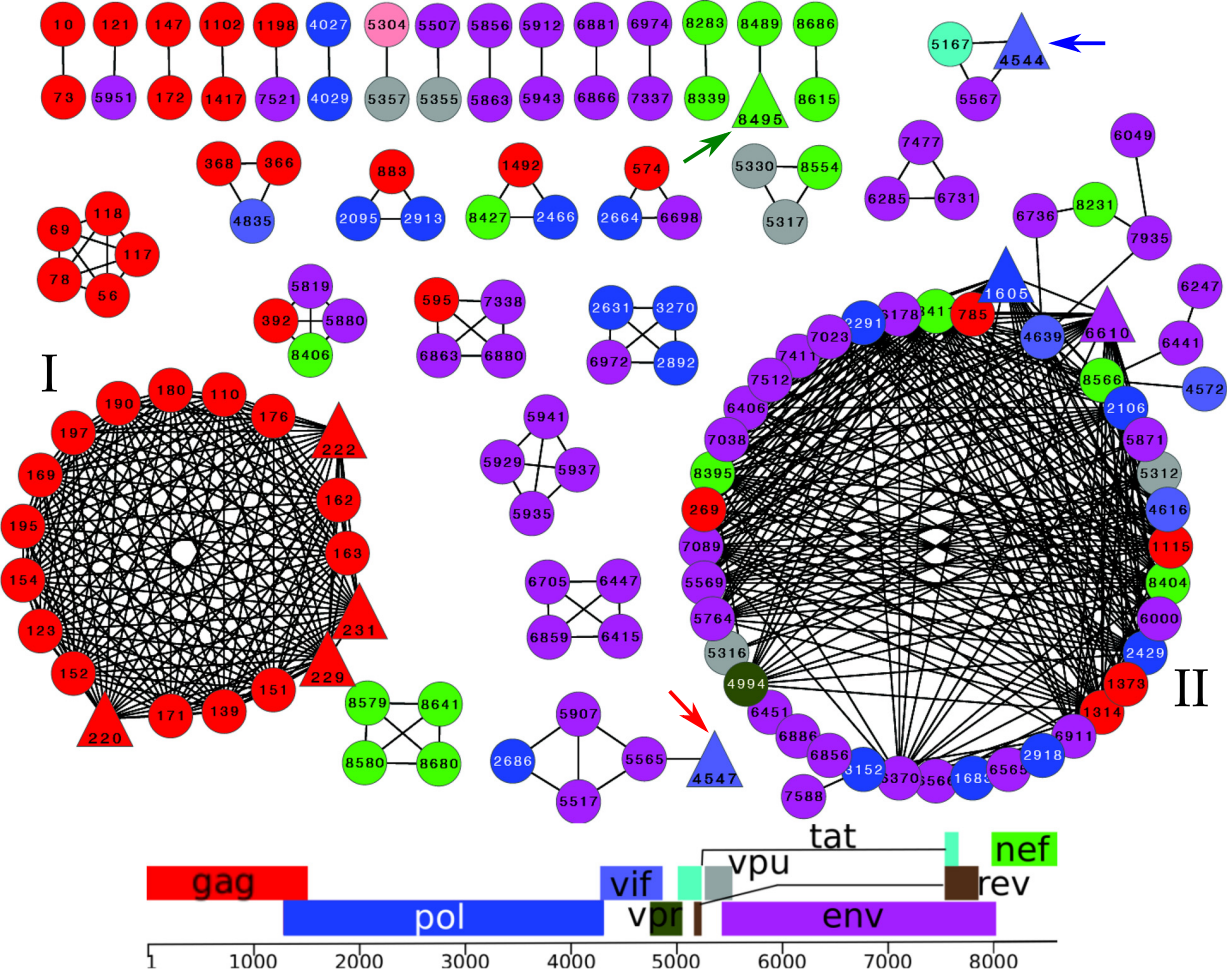
**Network of linked genomic sites. **Genomic sites with significant linkage disequilibrium are plotted in the network as nodes. An edge between two nodes represent that the two sites are linked. Sites belonging to different genes are shown in different colors: *gag* as red, *pol* as blue with white fonts, *vif* as violet, *vpr* as dark green, *tat* as light blue, *vpu* as grey, *env* as purple and *nef* as light green. No linked sites were found for *rev*. The legend shows the genomic position and colors assigned to the nine HIV-1 genes. Sites belonging to CTL epitope are shown as triangles. Green arrow indicates the Nef A24-RW8 epitope escape, and blue and red arrows indicate Vif B38-WI9 epitope escapes. “I” corresponds to the large cluster containing linked sites from the *gag* gene only, and “II” corresponds to the large cluster containing linked sites from several HIV-1 genes.

As a consequence of clonal interference, the fixation of genomes with high relative fitness due to the presence of beneficial epitope escape mutations, may result in genetic hitchhiking of other mutations present in the same haplotype [26,30,31]. The linkage disequilibrium between the nucleotide positions in HIV-1 are shown as a network of sites that are genetically linked in the set of all 30 haplotype genome sequences (Figure 7). Different colors represent different HIV-1 genes and triangles denote the mutations in epitopes (Figure 7). There are two major clusters of linked sites, one containing sites from the *gag* gene only (cluster I), and another containing sites from several genes (cluster II). In addition there are several clusters containing less than 5 sites. Epitope escapes are typically linked with one or more sites in non-epitope regions and these links could reflect compensatory mutations (Figure 7). Interestingly, the two early epitope escapes from the dominant CTL responses against Nef A24-RW8 (T5M genomic site: 8495, green arrow) and Vif B38-WI9 (genomic site: 4544 and 4547, blue and red arrow) were linked only with very few other sites (Figure 7). In contrast, the late epitope escapes from sub-dominant CTL responses like *pol* and *env* were genetically linked with several sites (cluster II, Figure 7).

The difference in linkage profiles can be explained by the homogeneity of the HIV-1 population at the start of infection (Figure 2B). The effect of genetic hitchhiking is clearly demonstrated by the large linkage cluster comprised of links between sites from *gag* (red) to *nef* (green) which probably have little or no functional linkage (cluster II, Figure 7). The hitchhiking mutations can be mildly advantageous or neutral [30], or may even be potentially deleterious [31]. For example, 22 non-synonymous mutations and 2 synonymous mutations (probably neutral) in the *env* gene have hitchhiked with 1 beneficial *env* epitope escape mutation (purple circles in cluster II, Figure 7). Thus, several non-epitope mutations hitchhike with immune escape mutations. The presence of genetic hitchhiking leading to multiple co-evolving genetic loci may influence the phylogenetic signal, especially if the number co-evolving loci is high. In our analysis we find that the phylogenetic signal for *env*, *gag* and *pol* genes, containing most of the genetically linked sites, is supported by strong bootstrap values at the late time point branches confirming that the topology is robust.

The haplotype reconstruction pipeline, using a combination of read clean-up and PredictHaplo, highlighting the importance of clonal interference and genetic hitchhiking. Our study provides the first evidence of clonal interference in HIV-1 genomes, and is one of the few demonstrating clonal interference in viral evolution. Our results indicate that CTL selection is not the only driving force shaping the evolution of escape mutations. Clonal interference and the genetic background of escape mutations both play an important role in viral evolution, and should be considered to calculate CTL selection pressures and HIV-1 escape rates [46]. Several studies have demonstrated that different epitopes escape mutations evolve in the same or in different epitopes and get fixated in the population. However, this can be a result of either genetic drift or selection or clonal interference. Using whole genome haplotypes, we show that clonal interference plays an essential role in the evolution and fixation of escape mutations.

Our study extends the scope of analysis that can be conducted using next-generation sequencing reads, and unraveled novel within-host evolutionary dynamics. Our pipeline is not limited by the sequencing technology, and the longer reads obtained using existing sequencing technologies, or new sequencing technologies like MiSeq (with 250 bp paired-end reads) and PacBio, can only improve the quality of haplotype reconstruction. The same approach can be applied to reconstruct haplotypes from MiSeq datasets (with 250 bp paired-end reads) by replacing the Roche specific read clean-up algorithm with a Illumina specific read clean-up algorithm. Henn et al. [11] provided assembled consensus sequences for each temporal sample which we used as reference sequences for our analysis. We performed the same analysis using the HXB2 reference sequence and found that this resulted in reduction of the number and quality of reconstructed haplotypes. Thus, proper read correction and clean-up is crucial for reconstructing global haplotypes.

A major drawback of this methodology may be that we miss low frequency recombinants in the population. Recombination during HIV-1 replication may facilitate the accumulation of multiple epitope escapes in single genomes. But during acute infection, the rate of recombination is known to be rather low [46-48], suggesting that most epitope escape mutations are sequentially acquired by the quasispecies [46]. Note that recombinant haplotypes that become sufficiently prevalent in the population will be detected as novel unique haplotypes. We simulated a population containing two genomes (each present at 44.5% frequency) taken from LHV simulations and generated a recombinant genome (present at 11% frequency). Three haplotypes corresponding to 3 true genomes were reconstructed at frequencies comparable to the true frequencies. The reconstructed recombinant genome had a Hamming distance of 25 from the true recombinant genome and both the reconstructed parent genomes had a Hamming distance of 20 from the true parent genomes, confirming that our pipeline can reconstruct recombinant genome prevalent in the population. Moreover, most of the errors were found in the first and last 20 bases similar to other control simulations (Table 2). Other simulations with higher prevalence (33% and 55% frequency) of recombinant genome lead to similar results. One possible way to detect presence of recombinants in reconstructed haplotypes would be to apply recombinant detection tools like recombinant identification program (RIP) [49], and SplitsTree [50]. Using RIP, we did not find any convincing evidence of recombination between day 476 haplotypes that might have given rise to any of the day 1543 haplotypes.

## Conclusions

We have shown that whole genome HIV-1 haplotypes can be reconstructed from short 454-sequencing reads with high fidelity. Reconstruction of genome haplotypes provides an opportunity to study the interaction between epitope escapes. Several epitope escapes evolve in single haplotypes, and different combinations of multiple epitope escapes become prevalent in the viral population. As a result of clonal interference, the fate of each escape mutation, also depends upon the fitness of other immune escape mutations prevalent in the viral population. The long range linkage disequilibrium between genomic sites suggests that clonal interference between HIV-1 genomes results in the fixation of several non-epitope mutations via genetic hitchhiking.

## Methods

### Simulated datasets generated for validation

To validate the haplotype reconstruction pipeline, we created multiple simulated HIV-1 populations. For each of these simulated datasets, we first generated 9 mutated haplotype genomes from a reference HIV-1 genome (GenBank accession number: JQ403055) by randomly selecting *r* sites such that *n*/2 ≤ *r *≤ *n*, where *n* represents either 2, 4 or 10% sites of the total number of sites. Three data sets U2, U4 and U10 (with varying nucleotide diversities of 2, 4 or 10%) were made with a uniform frequency distribution, i.e. all haplotypes were present at the same frequency (1/9) in the population. Three other data sets were made with a log-normal frequency distribution, with the same nucleotide diversities: 2, 4 and 10% for L2, L4 and L10, respectively. To implement a log-normal frequency distribution, some haplotype genomes were duplicated multiple times in the population to increase their frequencies. Thus, a typical population with a log-normal distribution consisted of 9 haplotypes present at the following copy numbers: 20, 12, 4, 2, 1, 1, 1, 1, 1, respectively. This generated a quasispecies with 5 minority haplotypes present at a frequency below 2.5*%*, and one dominant haplotype with a prevalence exceeding >45*%*. To model heterogeneity in mutation rate over HIV-1 genome, we added 3 hypervariable regions to genomes with 1% nucleotide diversity in the LHV data set. In the hypervariable regions, 50% of nucleotides had 10% nucleotide diversity, while the remaining sites had a 1% nucleotide diversity. We used a log-normal frequency distribution, similar to the L1 data set.

Additionally, we simulated 10 populations with a quasispecies structure. For each “quasispecies data set” (LQ4_i), we generated nine master genomes at a 4% diversity (similar to those above). After creating the 9 master genomes, we created mutated copies with at most 0.1*%* nucleotide diversity from the corresponding master sequence. This created a cloud of genomes around each master genome. The frequency of the nine quasispecies were distributed log-normally in the LQ4 dataset at the following copy numbers: 50, 24, 8, 4, 2, 2, 2, 2, 1 (as shown in Figure 1A).

We used the ART_454 software [42] to simulate 454 sequencing errors and generate *in silico* reads with an average coverage of 500 reads per base, mimicking the average depth obtained in Henn et al. [11]. The *in silico* reads obtained were analyzed using the haplotype reconstruction pipeline. A lower average coverage of 200 reads hardly changed the number of reconstructed haplotypes (results not shown).

### Longitudinal dataset used to study the within-host dynamics of HIV-1 haplotypes

The 454 sequence reads from six datasets V4137, V4136, V4139, V4140, V4676 and V4678 sampled from subject 9213 at day 0, 3, 59, 165, 476 and 1543 were downloaded from the NCBI SRA database [51]. As described in Henn et al. [11], the sequences were obtained by amplifying four overlapping PCR amplicons which span the entire protein coding region of HIV-1 genome. Subject 9213 was HLA-typed, and expressed the A01, A24, B38, B44 and Cw04 alleles. Henn et al. [11] recognized dominant and sub-dominant CTL responses against 7 HIV-1 epitopes in subject 9213 and described the immune escapes. We study the dynamics of these 7 escape mutations using the reconstructed haplotype genomes.

### Haplotype reconstruction pipeline

The haplotype reconstruction pipeline consists of two major steps: 1) Read clean-up, and 2) Prediction of haplotypes. Reads obtained from 454 NGS are known to contain three major types of process errors: a) carry forward and incomplete extension errors, b) homopolymer miscall errors, and c) InDels in non-homopolymer regions [11,36]. These 454 sequencer specific errors were corrected using the default parameter settings of ReadClean454 v1.0 (or RC454) software which uses *Mosaik* aligner [52] to align sequences to a reference genome sequence [11]. The RC454 cleaned reads were then subjected to PredictHaplo (version 0.5) [53] for haplotype reconstruction. We used the sample’s consensus assembly sequence obtained from Henn et al. [11], as the reference genome sequence for RC454 and PredictHaplo. We tested haplotype reconstruction at different parameter settings for PredictHaplo, and the default settings gave the most reliable results (results not shown). The cleaned-up reads obtained with the RC454 package were made compatible with PredictHaplo using customized Python scripts.

### Phylogenetic analysis

For phylogenetic analysis, genes and genomes from the reconstructed haplotypes were aligned using ClustalW [54]. The complete genome, *pol*, *env* and *gag* alignments, used in Figure 3, were 8878bp, 3040bp, 2645bp and 1546bp long, respectively. Phylogenetic trees were reconstructed using the maximum likelihood (ML) method performed by PHYML (version 3.0) [55]. ML analysis was conducted using the general time reversible (GTR) model, with six substitution rate categories for the gene phylogenies, and nine for the complete genome phylogeny, with the proportion of invariant sites set to 0. The gamma distribution parameter and nucleotide frequencies were estimated from the dataset for the phylogenetic reconstruction. From the parsimonious starting tree, the best tree generated using both the nearest neighbor interchange (NNI) and the subtree pruning and regrafting (SPR) approaches is shown in Figure 3. 100 bootstrap replicates were performed to assess the support for the branches and bootstrap support values ≥90 are indicated on the branches. For Figure 1A and Figure 2A, neighbor-joining phylogenetic trees were reconstructed using ClustalW because of their high sequence similarity [54]. To distinguish between synonymous and non-synonymous substitutions, we generated phylogenetic trees using MG and YAP codon substitution models performed by CodonPhyML [56]; however, the topology of the codon based phylogenetic trees was similar to the nucleotide trees.

### Selection and linkage analysis

To estimate the fitness and selection coefficients of mutated viral genomes, we used the method described by Maree et al. [22], which depends upon the frequency of wildtype and mutated viral genomes, and the time of evolution. To estimate selection coefficients, we considered that the frequency of a mutant haplotype rises from a value of 0.03 (frequency threshold below which we did not detect any haplotype) at *t*-1 to the observed frequencies at time *t* replacing its predecessor [57]. Note that the slow down of the selection coefficients is likely to be biased by the long intervals of late time points.

To detect the extent of positive selection, we first codon aligned the gene sequences using CodonAlign tool [58] and then used the random effects likelihood (REL) method from Datamonkey server [59,60]. Linkage disequilibrium between all polymorphic genomic sites was calculated using DNAsp and linkage values only significant after Bonferroni correction (p-value <0.4×10^-7^) were considered [61]. Linked sites were plotted as a network using Cytoscape (version 2.8) [62].

## Competing interests

The authors declare that they have no competing interests.

## Authors’ contributions

Designed and analyzed the experiments: AP and RDB. Performed the experiments: AP. Wrote the article: AP and RDB. Both authors read and approved the final manuscript.

## Supplementary Material

Additional file 1: Figure S1 Sequence alignments of the reconstructed haplotype and the corresponding quasispecies. Sequence alignment of four most prevalent haplotypes (A) haplotype_4, (B) haplotype_0, (C) haplotype_3, and (D) haplotype_5 and the corresponding quasispecies. Mismatches between the simulated genomes and the reconstructed haplotype are indicated as vertical lines with different colors corresponding to different nucleotides (A : Green, T : Red, G : Orange, C : Light blue and Gaps : Gray). The variation at each genomic site was present only in a subset of genomes demonstrating that the haplotypes are reconstructed as consensus sequences of the corresponding quasispecies. Click here for file

## References

[B1] Boutwell CL Rolland MM Herbeck JT Mullins JI Allen TM **Viral evolution and escape during acute HIV-1 infection** J Infect Dis 2010 202 Suppl 2 309 10.1086/655653 PMC2945609 20846038

[B2] Hill AL Rosenbloom DI Nowak MA **Evolutionary dynamics of HIV at multiple spatial and temporal scales** J Mol Med 2012 90 5 543 561 22552382 10.1007/s00109-012-0892-1 PMC7080006

[B3] Little SJ McLean AR Spina CA Richman DD Havlir DV **Viral dynamics of acute HIV-1 infection** J Exp Med 1999 190 6 841 850 10499922 10.1084/jem.190.6.841 PMC2195636

[B4] Salazar-Gonzalez JF Bailes E Pham KT Salazar MG Guffey MB Keele BF Derdeyn CA Farmer P Hunter E Allen S Manigart O Mulenga J Anderson JA Swanstrom R Haynes BF Athreya GS Korber BT Sharp PM Shaw GM Hahn BH **Deciphering human immunodeficiency virus type 1 transmission and early envelope diversification by single-genome amplification and sequencing** J Virol 2008 82 8 3952 3970 18256145 10.1128/JVI.02660-07 PMC2293010

[B5] McMichael AJ Borrow P Tomaras GD Goonetilleke N Haynes BF **The immune response during acute HIV-1 infection: clues for vaccine development** Nat Rev Immunol 2009 10 1 11 23 20010788 10.1038/nri2674 PMC3119211

[B6] Song H Pavlicek JW Cai F Bhattacharya T Li H Iyer SS Bar KJ Decker JM Goonetilleke N Liu MK Berg A Hora B Drinker MS Eudailey J Pickeral J Moody MA Ferrari G McMichael A Perelson AS Shaw GM Hahn BH Haynes BF Gao F **Impact of immune escape mutations on HIV-1 fitness in the context of the cognate transmitted/founder genome** Retrovirology 2012 9 1 1 14 23110705 10.1186/1742-4690-9-89 PMC3496648

[B7] Salazar-Gonzalez JF Salazar MG Keele BF Learn GH Giorgi EE Li H Decker JM Wang S Baalwa J Kraus MH Parrish NF Shaw KS Guffey MB Bar KJ Davis KL Ochsenbauer-Jambor C Kappes JC Saag MS Cohen MS Mulenga J Derdeyn CA Allen S Hunter E Markowitz M Hraber P Perelson AS Bhattacharya T Haynes BF Korber BT Hahn BH **Genetic identity, biological phenotype, and evolutionary pathways of transmitted/founder viruses in acute and early hiv-1 infection** J Exp Med 2009 206 6 1273 1289 19487424 10.1084/jem.20090378 PMC2715054

[B8] Fiebig EW Wright DJ Rawal BD Garrett PE Schumacher RT Peddada L Heldebrant C Smith R Conrad A Kleinman SH Busch MP **Dynamics of HIV viremia and antibody seroconversion in plasma donors: implications for diagnosis and staging of primary HIV infection** Aids 2003 13 1871 1879 12960819 10.1097/00002030-200309050-00005

[B9] Goonetilleke N Liu MK Salazar-Gonzalez JF Ferrari G Giorgi E Ganusov VV Keele BF Learn GH Turnbull EL Salazar MG Weinhold KJ Moore S CHAVI Clinical Core B Letvin N Haynes BF Cohen MS Hraber P Bhattacharya T Borrow P Perelson AS Hahn BH Shaw GM Korber BT McMichael AJ **The first T cell response to transmitted/founder virus contributes to the control of acute viremia in HIV-1 infection** J Exp Med 2009 206 6 1253 1272 19487423 10.1084/jem.20090365 PMC2715063

[B10] Mansky LM Temin HM **Lower in vivo mutation rate of human immunodeficiency virus type 1 than that predicted from the fidelity of purified reverse transcriptase** J Virol 1995 69 8 5087 5094 7541846 10.1128/jvi.69.8.5087-5094.1995 PMC189326

[B11] Henn MR Boutwell CL Charlebois P Lennon NJ Power KA Macalalad AR Berlin AM Malboeuf CM Ryan EM Gnerre S Zody MC Erlich RL Green LM Berical A Wang Y Casali M Streeck H Bloom AK Dudek T Tully D Newman R Axten KL Gladden AD Battis L Kemper M Zeng Q Shea TP Gujja S Zedlack C Gasser O **Whole genome deep sequencing of HIV-1 reveals the impact of early minor variants upon immune recognition during acute infection** PLoS Pathog 2012 8 3 1002529 10.1371/journal.ppat.1002529 PMC3297584 22412369

[B12] Ganusov VV de Boer RJ **Estimating costs and benefits of CTL escape mutations in SIV/HIV, infection** PLoS Comput Biol 2006 2 3 24 10.1371/journal.pcbi.0020024 PMC1420660 16604188

[B13] Kadolsky UD Asquith B **Quantifying the impact of human immunodeficiency virus-1 escape from cytotoxic t-lymphocytes** PLoS Comput Biol 2010 6 11 1000981 10.1371/journal.pcbi.1000981 PMC2973816 21079675

[B14] Althaus CL de Boer RJ **Dynamics of immune escape during HIV/SIV infection** PLoS Comput Biol 2008 4 7 1000103 10.1371/journal.pcbi.1000103 PMC2423483 18636096

[B15] Ganusov VV Goonetilleke N Liu MK Ferrari G Shaw GM McMichael AJ Borrow P Korber BT Perelson AS **Fitness costs and diversity of the cytotoxic T lymphocyte (CTL) response determine the rate of CTL escape during acute and chronic phases of HIV infection** J Virol 2011 85 20 10518 10528 21835793 10.1128/JVI.00655-11 PMC3187476

[B16] van Deutekom HWM Wijnker G de Boer RJ **The rate of immune escape vanishes when multiple immune responses control a HIV infection** J Immunol 2013 191 6 3277 3286 23940274 10.4049/jimmunol.1300962

[B17] Ganusov VV Neher RA Perelson AS **Mathematical modeling of escape of HIV from cytotoxic T lymphocyte responses** J Stat Mech Theor Exp 2013 2013 01 01010 10.1088/1742-5468/2013/01/P01010 PMC3961578 24660019

[B18] Liu MK Hawkins N Ritchie AJ Ganusov VV Whale V Brackenridge S Li H Pavlicek JW Cai F Rose-Abrahams M Treurnicht F Hraber P Riou C Gray C Ferrari G Tanner R Ping LH Anderson JA Swanstrom R CHAVI Core B Cohen M Karim SS Haynes B Borrow P Perelson AS Shaw GM Hahn BH Williamson C Korber BT Gao F **Vertical T cell immunodominance and epitope entropy determine HIV-1 escape** J Clin Invest 2013 123 1 380 23221345 10.1172/JCI65330 PMC3533301

[B19] Malim MH Emerman M **Hiv-1 sequence variation: Drift, shift, and attenuation** Cell 2001 104 4 469 472 11239404 10.1016/s0092-8674(01)00234-3

[B20] Lee HY Perelson AS Park S-C Leitner T **Dynamic correlation between intrahost HIV-1 quasispecies evolution and disease progression** PLoS Comput Biol 2008 4 12 1000240 10.1371/journal.pcbi.1000240 PMC2602878 19079613

[B21] da Silva J **The dynamics of HIV-1 adaptation in early infection** Genetics 2012 190 3 1087 1099 22209906 10.1534/genetics.111.136366 PMC3296244

[B22] Marée AF Keulen W Boucher CA de Boer RJ **Estimating relative fitness in viral competition experiments** J Virol 2000 74 23 11067 11072 11070001 10.1128/jvi.74.23.11067-11072.2000 PMC113186

[B23] Leviyang S **Computational inference methods for selective sweeps arising in acute HIV, infection** Genetics 2013 194 3 737 752 23666940 10.1534/genetics.113.150862 PMC3697977

[B24] Gray RR Salemi M Lowe A Nakamura KJ Decker WD Sinkala M Kankasa C Mulligan CJ Thea DM Kuhn L Aldrovandi G Goodenow MM **Multiple independent lineages of hiv-1 persist in breast milk and plasma** AIDS 2011 25 2 143 21173592 10.1097/QAD.0b013e328340fdaf PMC3032216

[B25] Feeney ME Tang Y Pfafferott K Roosevelt KA Draenert R Trocha A Yu XG Verrill C Allen T Moore C Mallal S Burchett S McIntosh K Pelton SI St. John MA Hazra R Klenerman P Altfeld M Walker BD Goulder PJR **HIV-1 viral escape in infancy followed by emergence of a variant-specific CTL response** J Immunol 2005 174 12 7524 7530 15944251 10.4049/jimmunol.174.12.7524

[B26] Strelkowa N Lässig M **Clonal interference in the evolution of influenza** Genetics 2012 192 2 671 682 22851649 10.1534/genetics.112.143396 PMC3454888

[B27] Łuksza M Lässig M **A predictive fitness model for influenza** Nature 2014 507 7490 57 61 24572367 10.1038/nature13087

[B28] Arjan JA de Visser M Zeyl CW Gerrish PJ Blanchard JL Lenski RE **Diminishing returns from mutation supply rate in asexual populations** Science 1999 283 5400 404 406 9888858 10.1126/science.283.5400.404

[B29] Miralles R Gerrish PJ Moya A Elena SF **Clonal interference and the evolution of RNA viruses** Science 1999 285 5434 1745 1747 10481012 10.1126/science.285.5434.1745

[B30] Lang GI Rice DP Hickman MJ Sodergren E Weinstock GM Botstein D Desai MM **Pervasive genetic hitchhiking and clonal interference in forty evolving yeast populations** Nature 2013 500 571 574 23873039 10.1038/nature12344 PMC3758440

[B31] Zanini F Neher RA **Deleterious synonymous mutations hitchhike to high frequency in HIV-1 env evolution** ArXiv e-prints 2013 1303.0805 10.1128/JVI.01529-13 PMC3807363 23986591

[B32] Fischer W Ganusov VV Giorgi EE Hraber PT Keele BF Leitner T Han CS Gleasner CD Green L Lo C-C Nag A Wallstrom TC Wang S McMichael AJ Haynes BF Hahn BH Perelson AS Borrow P Shaw GM Bhattacharya T Korber BT **Transmission of single HIV-1 genomes and dynamics of early immune escape revealed by ultra-deep sequencing** PloS one 2010 5 8 12303 10.1371/journal.pone.0012303 PMC2924888 20808830

[B33] Zagordi O Geyrhofer L Roth V Beerenwinkel N **Deep sequencing of a genetically heterogeneous sample: local haplotype reconstruction and read error correction** J Comput Biol 2010 17 3 417 428 20377454 10.1089/cmb.2009.0164

[B34] Schirmer M Sloan WT Quince C **Benchmarking of viral haplotype reconstruction programmes: an overview of the capacities and limitations of currently available programmes** Brief Bioinform 2012 15 3 431 442 23257116 10.1093/bib/bbs081

[B35] Töpfer A Zagordi O Prabhakaran S Roth V Halperin E Beerenwinkel N **Probabilistic inference of viral quasispecies subject to recombination** J Comput Biol 2013 20 2 113 123 23383997 10.1089/cmb.2012.0232 PMC3576916

[B36] Zagordi O Klein R Däumer M Beerenwinkel N **Error correction of next-generation sequencing data and reliable estimation of HIV quasispecies** Nucleic Acids Res 2010 38 21 7400 7409 20671025 10.1093/nar/gkq655 PMC2995073

[B37] Prosperi MC Salemi M **Qure: software for viral quasispecies reconstruction from next-generation sequencing data** Bioinformatics 2012 28 1 132 133 22088846 10.1093/bioinformatics/btr627 PMC3244773

[B38] Zagordi O Bhattacharya A Eriksson N Beerenwinkel N **Shorah: estimating the genetic diversity of a mixed sample from next-generation sequencing data** BMC Bioinformatics 2011 12 1 119 21521499 10.1186/1471-2105-12-119 PMC3113935

[B39] Prosperi MC Yin L Nolan DJ Lowe AD Goodenow MM Salemi M **Empirical validation of viral quasispecies assembly algorithms state-of-the-art and challenges** Sci Rep 2013 3 2837 24089188 10.1038/srep02837 PMC3789152

[B40] Burger H Weiser B Flaherty K Gulla J Nguyen P-N Gibbs RA **Evolution of human immunodeficiency virus type 1 nucleotide sequence diversity among close contacts** Proc Natl Acad Sci 1991 88 24 11236 11240 1763038 10.1073/pnas.88.24.11236 PMC53109

[B41] Keele BF Giorgi EE Salazar-Gonzalez JF Decker JM Pham KT Salazar MG Sun C Grayson T Wang S Li H Wei X Jiang C Kirchherr JL Gao F Anderson JA Ping LH Swanstrom R Tomaras GD Blattner WA Goepfert PA Kilby JM Saag MS Delwart EL Busch MP Cohen MS Montefiori DC Haynes BF Gaschen B Athreya GS Lee HY **Identification and characterization of transmitted and early founder virus envelopes in primary hiv-1 infection** Proc Natl Acad Sci 2008 105 21 7552 7557 18490657 10.1073/pnas.0802203105 PMC2387184

[B42] Huang W Li L Myers JR Marth GT **Art: a next-generation sequencing read simulator** Bioinformatics 2012 28 4 593 594 22199392 10.1093/bioinformatics/btr708 PMC3278762

[B43] Starcich BR Hahn BH Shaw GM McNeely PD Modrow S Wolf H Parks ES Parks WP Josephs SF Gallo RC Wong-Staal F **Identification and characterization of conserved and variable regions in the envelope gene of htlv-iii/lav, the retrovirus of aids** Cell 1986 45 5 637 648 2423250 10.1016/0092-8674(86)90778-6

[B44] Holmes EC Moya A **Is the quasispecies concept relevant to RNA viruses?** J Virol 2002 76 1 460 462 11739715 10.1128/JVI.76.1.460-462.2002 PMC135735

[B45] Grenfell BT Pybus OG Gog JR Wood JL Daly JM Mumford JA Holmes EC **Unifying the epidemiological and evolutionary dynamics of pathogens** Science 2004 303 5656 327 332 14726583 10.1126/science.1090727

[B46] Kessinger TA Perelson AS Neher RA **Inferring HIV escape rates from multi-locus genotype data** Front Immunol 2013 4 252 24027569 10.3389/fimmu.2013.00252 PMC3760075

[B47] Neher RA Leitner T **Recombination rate and selection strength in HIV intra-patient evolution** PLoS Comput Biol 2010 6 1 1000660 10.1371/journal.pcbi.1000660 PMC2813257 20126527

[B48] Batorsky R Kearney MF Palmer SE Maldarelli F Rouzine IM Coffin JM **Estimate of effective recombination rate and average selection coefficient for HIV in chronic infection** Proc Natl Acad Sci 2011 108 14 5661 5666 21436045 10.1073/pnas.1102036108 PMC3078368

[B49] Siepel AC Halpern AL Macken C Korber BT **A computer program designed to screen rapidly for hiv type 1 intersubtype recombinant sequences** AIDS Res Hum Retroviruses 1995 11 11 1413 1416 8573400 10.1089/aid.1995.11.1413

[B50] Huson DH Bryant D **Application of phylogenetic networks in evolutionary studies** Mol Biol Evol 2006 23 2 254 267 16221896 10.1093/molbev/msj030

[B51] **Sequence Read ArcHIVe (SRA)** [http://www.ncbi.nlm.nih.gov/Traces/sra]

[B52] **MOSAIK** [http://code.google.com/p/mosaik-aligner/]

[B53] **PredictHaplo** [http://bmda.cs.unibas.ch/HivHaploTyper/]

[B54] Thompson JD Gibson TJ Higgins DG **Multiple sequence alignment using ClustalW and ClustalX** Curr Protoc Bioinformatics 2002 2.3 1 22 10.1002/0471250953.bi0203s00 18792934

[B55] Guindon S Gascuel O **A simple, fast, and accurate algorithm to estimate large phylogenies by maximum likelihood** Syst Biol 2003 52 5 696 704 14530136 10.1080/10635150390235520

[B56] Gil M Zanetti MS Zoller S Anisimova M **Codonphyml: fast maximum likelihood phylogeny estimation under codon substitution models** Mol Biol Evol 2013 30 6 1270 1280 23436912 10.1093/molbev/mst034 PMC3649670

[B57] **Estimating relative fitness from competition experiments** [http://bioinformatics.bio.uu.nl/rdb/fitness.html] 10.1128/jvi.74.23.11067-11072.2000 PMC113186 11070001

[B58] **CodonAlign** [http://www.hiv.lanl.gov/content/sequence/CodonAlign/codonalign.html]

[B59] Pond SLK Frost SD **Not so different after all: a comparison of methods for detecting amino acid sites under selection** Mol Biol Evol 2005 22 5 1208 1222 15703242 10.1093/molbev/msi105

[B60] Delport W Poon AF Frost SD Pond SLK **Datamonkey 2010: a suite of phylogenetic analysis tools for evolutionary biology** Bioinformatics 2010 26 19 2455 2457 20671151 10.1093/bioinformatics/btq429 PMC2944195

[B61] Librado P Rozas J **DnaSP v5: a software for comprehensive analysis of dna polymorphism data** Bioinformatics 2009 25 11 1451 1452 19346325 10.1093/bioinformatics/btp187

[B62] Smoot ME Ono K Ruscheinski J Wang P-L Ideker T **Cytoscape 2.8: new features for data integration and network visualization** Bioinformatics 2011 27 3 431 432 21149340 10.1093/bioinformatics/btq675 PMC3031041

