# Supplementary material for: Reliable reconstruction of HIV-1 whole genome haplotypes reveals clonal interference and genetic hitchhiking among immune escape variants
Source: Retrovirology. 2014 Jul 4;11:56. doi: 10.1186/1742-4690-11-56 (PMC4227095; doi:10.1186/1742-4690-11-56)
Supplement: Additional file 1: Figure S1 — Sequence alignments of the reconstructed haplotype and the corresponding quasispecies. Sequence alignment of four most prevalent haplotypes (A) haplotype_4, (B) haplotype_0, (C) haplotype_3, and (D) haplotype_5 and the corresponding quasispecies. Mismatches between the simulated genomes and the reconstructed haplotype are indicated as vertical lines with different colors corresponding to different nucleotides (A : Green, T : Red, G : Orange, C : Light blue and Gaps : Gray). The variation at each genomic site was present only in a subset of genomes demonstrating that the haplotypes are reconstructed as consensus sequences of the corresponding quasispecies. [file 1742-4690-11-56-S1.pdf]

Additional File 1

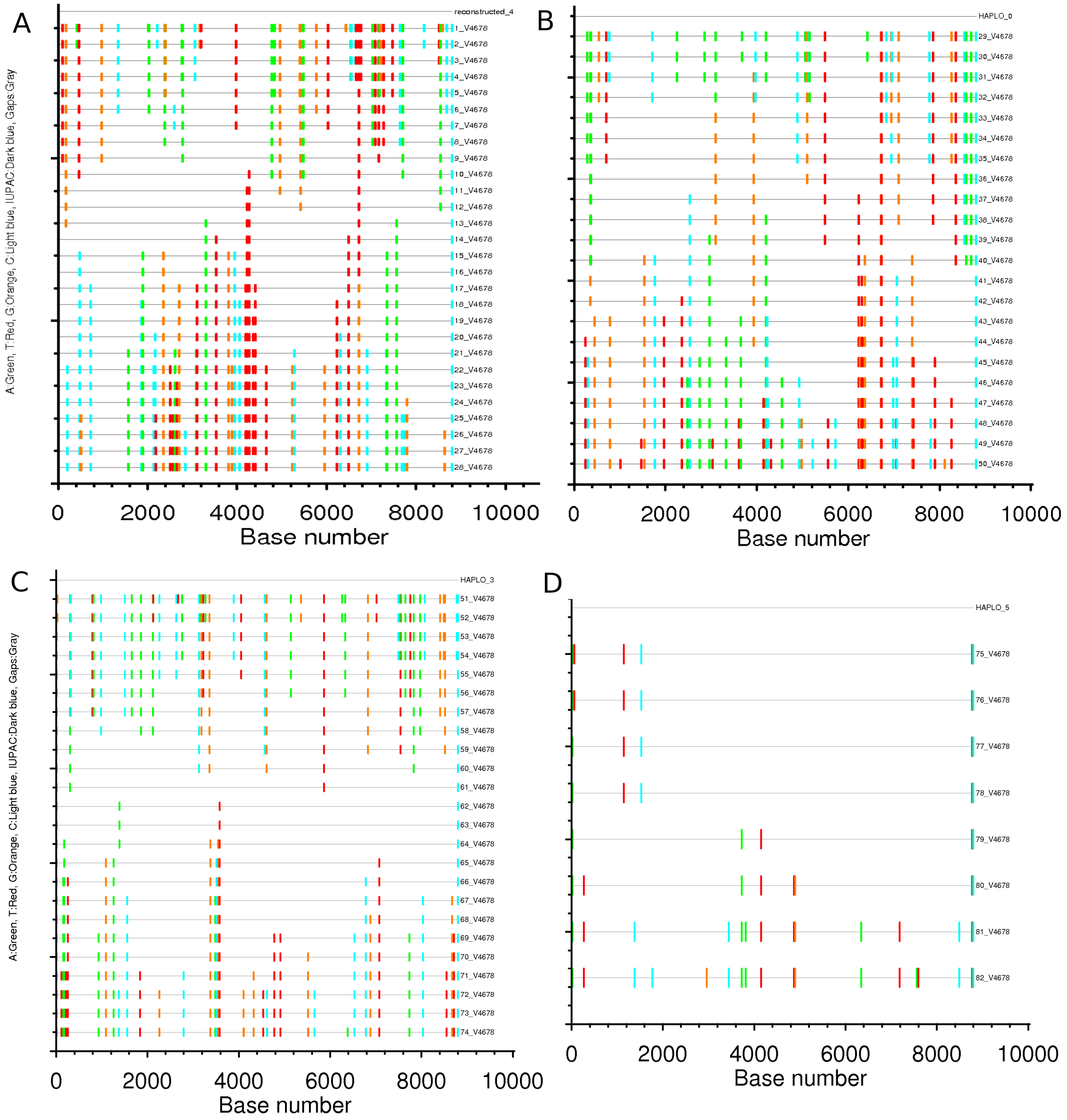

Figure S1: Sequence alignments of the reconstructed haplotype and the corresponding quasispecies. Sequence alignment of four most prevalent haplotypes (A) haplotype_4, (B) haplotype_0, (C) haplotype_3, and (D) haplotype_5 and the corresponding quasispecies. Mismatches between the simulated genomes and the reconstructed haplotype are indicated as vertical lines with different colors corresponding to different nucleotides (A: Green, T: Red, G: Orange, C: Light blue and Gaps: Gray). The variation at each genomic site was present only in a subset of genomes demonstrating that the haplotypes are reconstructed as consensus sequences of the corresponding quasispecies.
